# Gingival Mesenchymal Stem/Progenitor Cells: A Unique Tissue Engineering Gem

**DOI:** 10.1155/2016/7154327

**Published:** 2016-05-29

**Authors:** Karim M. Fawzy El-Sayed, Christof E. Dörfer

**Affiliations:** ^1^Clinic of Conservative Dentistry and Periodontology, Christian Albrechts University of Kiel, Arnold Heller Straße 3, Haus 26, 24105 Kiel, Germany; ^2^Oral Medicine and Periodontology Department, Faculty of Oral and Dental Medicine, Cairo University, Egypt

## Abstract

The human gingiva, characterized by its outstanding scarless wound healing properties, is a unique tissue and a pivotal component of the periodontal apparatus, investing and surrounding the teeth in their sockets in the alveolar bone. In the last years gingival mesenchymal stem/progenitor cells (G-MSCs), with promising regenerative and immunomodulatory properties, have been isolated and characterized from the gingival lamina propria. These cells, in contrast to other mesenchymal stem/progenitor cell sources, are abundant, readily accessible, and easily obtainable via minimally invasive cell isolation techniques. The present review summarizes the current scientific evidence on G-MSCs' isolation, their characterization, the investigated subpopulations, the generated induced pluripotent stem cells- (iPSC-) like G-MSCs, their regenerative properties, and current approaches for G-MSCs' delivery. The review further demonstrates their immunomodulatory properties, the transplantation preconditioning attempts via multiple biomolecules to enhance their attributes, and the experimental therapeutic applications conducted to treat multiple diseases in experimental animal models in vivo. G-MSCs show remarkable tissue reparative/regenerative potential, noteworthy immunomodulatory properties, and primary experimental therapeutic applications of G-MSCs are very promising, pointing at future biologically based therapeutic techniques, being potentially superior to conventional clinical treatment modalities.

## 1. Introduction

The human periodontium, the tooth supporting and investing organ, comprising the alveolar bone, the periodontal ligament, the root cementum, and the gingiva develops and functions as one unit. The majority of the periodontal tissues originate embryonically from the neural crest ectomesenchyme [1]. The gingiva, histologically composed of epithelium and connective tissue, constitutes a distinctive as well as a pivotal component of the human periodontium developmentally and anatomically, surrounding the necks of the teeth and investing the tooth-bearing alveolar bone. One of the gingiva's renowned characteristics is its notable wound healing and regenerative aptitude, with a fast reconstitution of tissue architecture following injury or excision with little, if any, evidence of scarring [2]. This tissue is easily accessible and is often resected during standard surgical procedures, including dental crown lengthening and multiple periodontal surgeries, with minimal discomfort to the patient [3].

**Figure 1:**
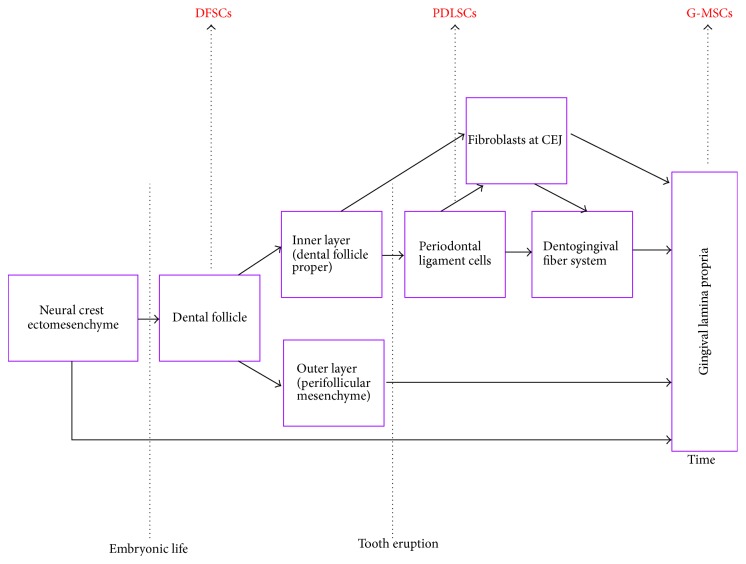
Schematic drawing of the oral tissues contributing to the developmental origin of human gingival lamina propria. DFSCs: dental follicle stem cells, G-MSCs: gingival mesenchymal stem/progenitor cells, PDLSCs: periodontal ligament stem cells.

Developmentally, the craniofacial ectomesenchyme is derived from the neural crest and the mesoderm. The multipotent cranial neural crest cells (CNCCs) migrate ventrolaterally to reside in the first branchial arches, starting from the four-somite stage, giving rise to mesenchymal structures in the craniofacial region, including neural tissues, cartilage, bone, and teeth [4, 5]. In addition to a common neural crest ectomesenchymal origin, lined by ectoderm for all oral soft tissues, the tooth-investing gingival connective tissue shows a unique developmental origin, arising partly from the perifollicular mesenchyme (the outer layer of the dental follicle) [1], as well as partly from the dental follicle proper (the inner layer of the dental follicle) [6], from which dental follicle stem/progenitor cells (DFSCs) were isolated [7]. Periodontal ligament cells [8], originating themselves from the dental follicle proper [1] and from which a subpopulation of periodontal ligament stem/progenitor cells (PDLSCs) has been characterized [9], further contribute to its development. In addition, earlier studies demonstrated the presence of fibroblasts stemming from the inner layer of the dental follicle in the free gingival lamina propria at the cementoenamel junction [6] and further suggested that the dentogingival fiber system originates in part from the periodontal ligament cells [8] (Figure 1). This developmental contribution, provided by the dental follicle proper and the periodontal ligament cells to the perifollicular mesenchyme, accounts for an anatomical distinctiveness of the tooth-investing gingival connective tissue compared to other oral mucosal tissues [3].

**Figure 2:**
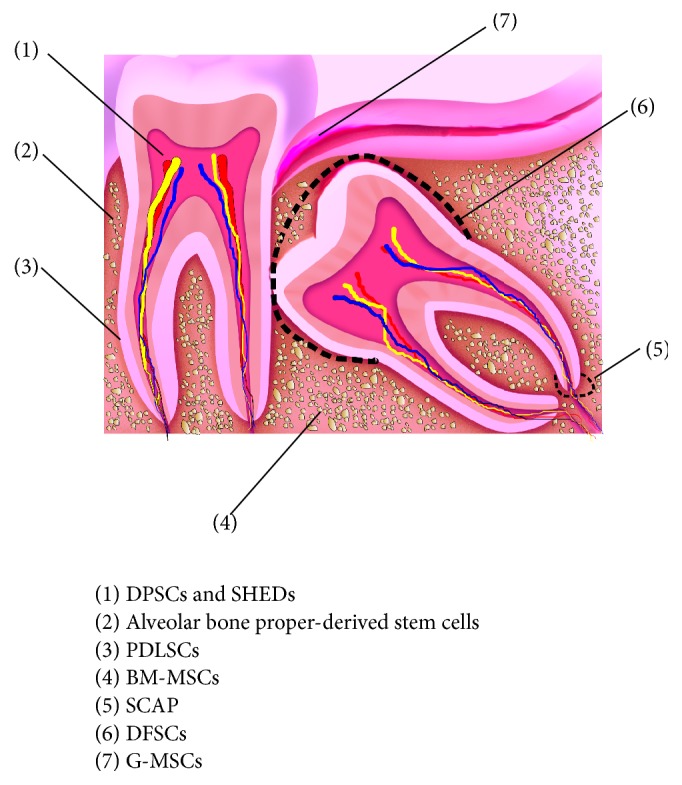
Sources of oral stem/progenitor cells isolated. DFSCs: dental follicle stem cells, G-MSCs: gingival mesenchymal stem/progenitor cells, PDLSCs: periodontal ligament stem cells, SHEDs: stem cells from the human exfoliated deciduous teeth, DPSCs: dental pulp stem cells, BM-MSCs: bone marrow mesenchymal stem cells, and SCAP: stem cells from the apical papilla.

The numerous functions of adult gingival wound fibroblasts and their variance in responsiveness to growth factors as well as their capacity to produce particular extracellular matrix proteins during healing validated an earlier hypothesis that gingival connective tissue fibroblasts embody a heterogeneous cell population [8, 10–13]. It further implied the existence of a resident population of adult mesenchymal stem/progenitor cells, giving rise to these heterogeneous cells. Previous studies described the isolation of progenitors from oral soft tissues, including the incisive papillae and rugae area of the palate [14], the maxillary tuberosity [15], the oral mucosa [16], the whole [17], the attached and free [3, 18, 19], and the hyperplastic gingiva [20]. Clinically, special attention is placed on the gingiva as a source mesenchymal stem/progenitor cells, representing the most abundant, accessible, and conservative minimally invasive source for stem/progenitor cells' isolation from the oral cavity [21] (Figure 2).

## 2. Gingival Mesenchymal Stem/Progenitor Cells' (G-MSCs) Isolation

A wide array of designations currently exist for mesenchymal stem/progenitor cells isolated from the gingival lamina propria, including gingiva-derived mesenchymal stem/stromal cells (G-MSCs) [22], gingival-tissue-derived stem cells (GT-MSCs) [18], gingival multipotent progenitor cells (GMPCs) [17], and gingival margin-derived stem/progenitor cells [3]. For clarity, the term gingival mesenchymal stem/progenitor cells (G-MSCs) will be used to uniformly designate these cells in the present review.

Studies reporting on techniques for G-MSCs' isolation surgically obtained gingival tissue samples from human subjects or animals via gingivectomy techniques and deepithelialized them, leaving only the connective tissue. The connective tissue biopsies were minced and digested to obtain single-cell suspensions [18, 20, 23–25] or kept intact and the tissue explants culture method was used to grow out the adherent connective tissue cells [3, 15, 26, 27]. The obtained cells were subsequently cultured and expanded in vitro for 3-4 weeks.

Diverse G-MSCs' isolation and expansion protocols were proposed (Table 1). Some of the outlined protocols, except for I, III, IV, and V, did not attempt to select stem/progenitor cells' population from the heterogeneous gingival connective tissue cells via single-cell cloning [23, 28, 29] or magnetic activated cell sorting (MACS) techniques [3, 30]. This raises a question about whether thereafter characterized cultures would represent enriched mesenchymal stem/progenitor cell cultures or merely mixed gingival connective tissue cell cultures, encompassing stem/progenitor cells in their original low percentages, usually present in the gingival lamina propria. A recent study relying on a STRO-1/MACS scheme for G-MSCs' isolation underlined the importance of the utilization of a cell selection/sorting technique for G-MSCs' isolation, pointing out that two cell populations, a STRO-1/MACS^+^ and a STRO-1/MACS^−^, with distinctive properties and marker expression profiles exist in the human gingival connective tissue. The study demonstrated that the STRO-1/MACS^+^-cell population, in contrast to the STRO-1/MACS^−^ one, harbored the cells with stem/progenitor cells' characteristics and distinctive osteogenic marker expression and validated thereby the effectiveness of the STRO-1/MACS technique in the field of G-MSCs' isolation [3].

## 3. G-MSCs' Characterization

To characterize G-MSCs and compare their properties to bone marrow mesenchymal stromal cells (BM-MSCs), the forerunner and gold standard in the field mesenchymal stromal cells' (MSCs) isolation, characterization, and research [31], most studies referenced the minimal criteria proposed by the International Society for Cellular Therapy (ISCT) for MSCs' characterization [32]. MSCs should show self-renewal capabilities and plastic adherence under standard culture conditions. More than 95% of the alleged MSCs' population should express the surface markers CD73, CD90, and CD105, as measured by flow cytometry, and these cells must lack the expression (less than 2%) of the surface markers CD11b, CD14, CD19, CD34, CD45, CD79*α*, and HLA-DR. Finally, the cells should show the ability to differentiate into at least three tissue lineages (e.g., osteoblastic, adipocytic, and chondroblastic) under standard in vitro inductive conditions.

### 3.1. Self-Renewal

Self-renewal ability is one of the basic cellular characteristics of stem/progenitor cells. MSCs may divide asymmetrically, giving rise to two distinct daughter cells, one MSC and a second daughter programmed to differentiate into a committed lineage, or divide symmetrically, producing two identical copies of the original MSC [44]. Similarly, human G-MSCs demonstrated this ability through the formation of colony forming units (CFUs) [3, 15, 17, 18, 20, 22, 25].

As compared to BM-MSCs, G-MSCs show a faster proliferation rate (the population doubling time remaining constant in the range of 30–50 hours from primary to long-term cultures, whereas in BM-MSCs it increases from 50–60 hours in primary to up to 160–180 hours in long-term cultures) [18, 20, 25]. This significant property was primarily ascribed to a continuous activation of the telomerase enzyme even in long-term cultures [25]. Unlike BM-MSCs, which demonstrate abnormalities typical of the Hayflick model of cellular aging [45] at 8–10 passages, G-MSCs retain a stable morphology, maintain normal karyotype, do not lose MSCs' characteristics at higher passages, and are not tumorigenic [15, 18], despite their origin from healthy [3] or hyperplastic/inflamed gingival tissue [20, 23].

### 3.2. Multilineage Differentiation Potential

Similar to previous investigations on MSCs from other tissue sources, several studies reported on a multilineage differentiation ability of G-MSCs into osteoblastic, adipocytic, chondrocytic, endothelial, and neural directions, when incubated in in vitro inductive culture conditions (Table 2) [15, 17, 20, 22, 25, 40].

Osteogenic differentiation was demonstrated by the formation of calcified Alizarin-Red positive deposits [3, 15, 17, 18, 20, 22, 25] and through transmission electron microscopic (TEM) ultrastructural examinations, showing cellular features of mature osteoblasts, including the presence of two or three extended nucleoli, mitochondria with extended morphology, vacuoles in the process of exocytosis, extracellular granular and nongranular matrix, collagen fibers, and areas of early mineralization [46]. Osteogenic differentiation was further demonstrated on the mRNA level through the expression of bone specific markers, including Runx2, collagen I, collagen III, alkaline phosphatase (ALP), osteonectin (ON), osteopontin (OP), and osterix [3, 25, 28, 39]. G-MSCs with suitable carriers implanted subcutaneously into immunocompromised mice generated connective tissue-like structures [20, 25], bone matrix [17, 22, 47], or mineralized tissues that exhibited certain similarities to cementum and bone, positively staining for collagen (Col), Ca, cementum attachment protein (CAP), cementum protein 1 (CP-1), bone sialoprotein (BSP), ALP, and osteocalcin (OC) [48].

Adipogenic differentiation was demonstrated by Oil-Red-O staining and the expression of the adipogenic markers peroxisome proliferator-activated receptor gamma (PPAR*γ*), fatty acid synthase, and lipoprotein lipase (LPL) [3, 18, 25, 28]. Alginate-encapsulated G-MSCs were able to be differentiated into osteogenic and adipogenic tissues in vitro and through scanning electron microscopic (SEM) examinations demonstrated the formation of hydroxyapatite-like crystalline structures [47].

Chondrogenic differentiation was evident by Toluidine-Blue staining and the expression of Sox-9, aggrecan, and Col-II [18] or by Alcian blue staining and aggrecan expression [3, 49] in 3D micromasses of G-MSCs. In a study, G-MSCs cultured for 3 weeks in chondrogenic inductive medium, followed by 2 weeks of hypoxic conditioning to induce cellular hypertrophy, further demonstrated Sox-9-dependent differentiation into chondrocyte and synoviocyte lineages in self-organized distinct areas that resembled native cartilage templates. Nonhypoxic conditions induced the expression of Sox-9, aggrecan, and Col-IIA1. With hypoxia cellular hypertrophy was induced, with downregulation of Sox-9, aggrecan, and Col-IIA1 and upregulation of Indian Hedgehog (IHH), Col-XA1, vascular endothelial growth factor a (VEGFA), matrix metalloproteinase 13 (MMP13), Runx2, and Col-IA1. Peripheral cells in the micromass cultures were organized in layers of cuboidal cells with villous structures facing the inductive medium and were strongly positive for cadherin-11, a marker of synoviocytes. Inhibition of cadherin-11 by siRNA transfection showed inhibition of the formation of this peripheral cell lining [49].

A further study reported on the ability of G-MSCs for neuronal and endothelial differentiation [25]. This remains however to be a controversial issue in the scientific community, regarding the minimal evidence provided to support the differentiation results. The study reported that neuronal differentiation was evident by the immunohistochemical staining of glial fibrillary acidic protein (GFAP), neurofilament 160/200 (NF-M), and *β*-tubulin III in the neuronal induced cultures. Here it should be noted that GFAP is not specific for neuronal differentiation, as the protein filament, aside of being expressed by astrocytes [50] and ependymal cells [51], is present in many cell types including glomeruli and peritubular fibroblasts in rat kidneys [52], Leydig cells of the testis in humans [53], and human osteocytes and chondrocytes [54]. For endothelial differentiation, the study relied solely on the expression of CD31 [25]. Apart from the immunohistochemical staining, no quantification of specific gene expressions for neuronal or endothelial differentiation was undertaken.

### 3.3. MSCs' Associated Markers

Currently, no explicit surface marker constellation exists for MSCs' characterization. For standardization purposes, studies commonly refer to the marker arrangement proposed by the ISCT [32] for G-MSCs' identification (see the above). Many studies further augmented the ISCT's list by additional markers, including CD13, CD38, CD44, CD54, CD117, CD144, CD146, CD166, Sca-1, STRO-1, SSEA-4, Oct-3/4, Oct-4A, Nanog, nestin, integrin *β*1, and vimentin [3, 24, 26, 36, 37, 43] (most commonly explored markers listed in Table 3).

Marker expression was shown to be altered by culturing conditions, where G-MSCs cultured as 3D spheroids demonstrated elevated expression Stro-1, CXC chemokine receptor 4 (CXCR-4), Oct-4, and Nanog, important transcriptional factors relevant to stem cell properties, and decreased expression of other MSCs-associated markers, including CD29, CD90, and CD105 [34]. Ascorbic acid (vitamin C) primed G-MSCs significantly elevated the expression of SSEA-3, Sox-2, Oct-3/4, Nanog, and TRA-1-60 [27]. Oct-3/4, Nanog, and Sox-2 expression are vital for maintaining a progenitor status with an unlimited stem cells' division, without affecting their self-renewal or differentiation capacity [55, 56]. Nanog is further a key gene for maintaining the cells' pluripotency [55, 57]. The expression of pluripotency markers, including Oct-3/4, Nanog, and Sox-2, by G-MSCs, similar to the expression described in a population of dental pulp pluripotent-like stem cells (DPPSCs) [55], presents an interesting finding and questions the true potential of G-MSCs. A proposed explanation, similar to previously described stem/progenitor cell sources [58, 59] could be that the human gingiva harbors subpopulations of stem/progenitor cells with pluripotent characteristics. However, another and in our view very interesting explanation is that pluripotency could be maintained/induced through specific culture conditions or biomolecules. DPPSCs cultured in a cell culture medium containing LIF (leukemia inhibitory factor), EGF (epidermal growth factor), and PDGF (platelets derived growth factor) expressed the pluripotency markers [55]. Similarly, G-MSCs' incubation in ascorbic acid (see the following) significantly elevated their pluripotency markers [27].

This varied expression of multi- as well as pluripotent markers by G-MSCs under different settings/culturing conditions, their remarkable differentiation potential (even apparently breaching endodermal and neuroectodermal barriers), and their long-term telomerase expression [25], similar to embryonic stem cells, raise the question about whether the true potential of G-MSCs has been elucidated yet. Further extensive research is needed in this area to precisely define the genuine potential of G-MSCs, the possible presence of subpopulations with diverse differentiation potentials, and the development of culture techniques and settings that could positively influence/direct their cellular properties prior to transplantation.

## 4. G-MSCs' Subpopulations

A study demonstrated the existence of G-MSCs in inflamed gingival tissues, exhibiting a phenotypic profile, an in vitro differentiation capacity and an in vivo developmental potential similar to G-MSCs obtained from healthy gingival tissues [23]. This finding is of prime importance, as G-MSCs isolated from the gingival tissues usually reside in a field of constant bacterial challenge, with resultant tissue inflammatory changes, in the oral cavity. It further underlines their positive attributes. Their resistance to inflammatory stimuli while retaining their MSCs' properties makes them a promising cellular source for tissue engineering therapeutic applications in vivo, where they could be exposed to similar inflammatory conditions.

It was further demonstrated that the gingival lamina propria contains two subpopulations of G-MSCs: 90% neural-crest-derived G-MSCs (N-GMSCs) and 10% mesoderm-derived G-MSCs (M-GMSCs) with distinctive stem cell properties. Compared to M-GMSCs, N-GMSCs showed an elevated aptitude to differentiate into neural cells, as was evident by an increase in nestin, neurofilament M (NF-09), and *β*-tubulin III expression, as well as chondrocytes, as was evident by Col-II and Sox-9 expression, and demonstrated enhanced immunomodulatory properties, inducing activated T-cell apoptosis, elevation of Tregs, and downregulation of Th-17. It appeared that the N-GMSCs mediated immunomodulation is associated with an elevated expression of Fas Ligand (FasL). However, both subpopulations showed no difference in their aptitude for osteogenic and adipogenic differentiation [36]. Further studies are needed to investigate the presence and properties of additional G-MSCs' subpopulations.

## 5. Gingiva-Derived iPSCs

The encouraging therapeutic prospective/potential of MSCs in the field of tissue engineering and regenerative approaches has highlighted the need for identifying easily accessible sources to obtain them in large quantities. A proposed source for obtaining large populations of MSCs is through the controlled induction of pluripotent stem cells (iPSCs) from the abundant and readily accessible human gingival fibroblasts (GFs).

Initially, iPSCs were generated from human and mouse GFs via genomic insertion of reprogramming factors carried on retroviral vectors [60, 61]. Although currently retroviral vectors provide the highest transfection efficiency, the technique harbors a high risk of cellular genetic mutation and viral genomic transmission [62, 63]. Retroviral-induced iPSCs from the GFs showed fast proliferation with a typical fibroblastic morphology and, unlike classical iPSCs cultures, the capacity to proliferate on standard culture flasks in the absence of a feeder cell layer. The gingival iPSCs, generated through transduction of Oct-3/4, Sox-2, Klf4, and c-myc and subsequently cultured for two weeks and passaged (up to 5–10 passages) in a MSCs' medium, consisting of minimum essential medium eagle-alpha modified (*α*-MEM) with 10% fetal calf serum (FCS), penicillin/streptomycin, sodium pyruvate, L-ascorbate-2-phosphate, L-glutamine, nonessential amino acids and HEPES, expressed MSCs-associated markers (CD73, CD90, CD105, CD146, and CD166), lacked the expression of the pluripotent (TRA160, TRA181, and ALP) and hematopoietic markers (CD14, CD34, and CD45), and showed a multilineage differentiation potential into osteoblastic, adipocytic, and chondrocytic directions [64]. The lack of pluripotent markers' expression however questions whether the described cells are true iPSCs or if they have undergone differentiation under the MSCs' culture conditions into a mesenchymal stromal cell type and qualify them, therefore, to be designated “iPSCs-like G-MSCs.”

In a second study, true iPSCs were generated from GFs through a virus/integration-free and feeder-free approach, delivering the reprogramming factors of Oct-4, Sox-2, Klf4, L-myc, Lin28, and TP53 shRNA on episomal plasmid vectors. The generated gingival iPSCs presented morphology and proliferation characteristics similar to embryonic stem cells (ESCs), expressed, in contrast to the earlier study [64], pluripotent markers including Oct-4, Tra181, Nanog, and SSEA-4, maintained a normal karyotype, and showed decreased CpG methylation ratio in the promoter regions of Oct-4 and Nanog. In vivo teratoma formation assay demonstrated the development of tissues representative of the three germ layers, confirming their pluripotency [43].

A further study demonstrated in an opposite direction the successful differentiation of GFs integration-free episomal plasmid vectors-derived iPSCs into CD44^+^CD73^+^CD90^+^CD105^+^ G-MSCs-like cells, with osteogenic, adipogenic, and chondrogenic differentiation capabilities [65]. A recent study tested the osteogenic differentiation of iPSCs from GFs seeded on a nanohydroxyapatite/chitosan gelatin (nHA/CG) porous scaffold with two shapes (rod and sphere) in vitro and in vivo. Results revealed that sphere-nHA/CG significantly increased iPSCs proliferation and their osteogenic differentiation aptitude in vitro. iPSCs which were cultured on sphere-nHA/CG produced large, while iPSCs which were grown on rod-nHA/CG showed tiny bone in-vivo [66]. These results point clearly again at the influential effect of culturing conditions/matrix properties on the cellular differentiation potential.

## 6. Immunomodulatory Properties of G-MSCs

**Figure 3:**
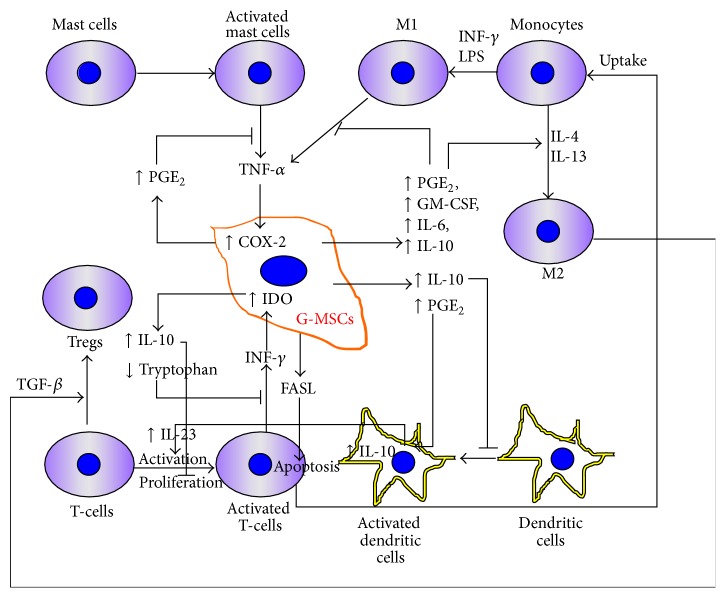
Immunomodulatory “crosstalk” between G-MSCs and mast cells, macrophages (with their M1 and M2 phenotypes), dendritic cells, and T-cells. COX-2: cyclooxygenase-2; PGE_2_: prostaglandin E_2_; GM-CSF: granulocyte-macrophage colony-stimulating factor; INF: interferon; IL: interleukin; TGF: transforming growth factor; IDO: indoleamine 2,3-dioxygenase; LPS: lipopolysaccharide.

Besides the well-established self-renewal, multipotent differentiation, and tissue regeneration capabilities, G-MSCs, similar to other MSCs sources, possess outstanding immunomodulatory properties, which could be of great therapeutic interest. Generally, MSCs are nonimmunogenic and hold immunomodulatory capability, allowing for their allogenic transplantation without host immunosuppression. The interaction that occurs between G-MSCs and the surrounding inflammatory cells is thereby very complex (Figure 3). These immunomodulatory properties allow G-MSCs to ameliorate inflammatory diseases therapeutically, through their influence on the local microenvironment [33]. The cellular and molecular mechanisms, by which G-MSCs exert their immunomodulatory effects, are currently a matter of intense research, representing a potentially promising tool in cellular therapy [15].

## 7. Effects of G-MSCs on the Innate Immune System

The innate immune system is the first line of the host's defense and is comprised of several types of immune molecules and cells [67], particularly toll-like receptors (TLRs), dendritic cells (DCs), macrophages, and mast cells (MCs). Multiple studies revealed how G-MSCs exhibit potent immunomodulatory effects on these cells [29, 68].

### 7.1. Toll-Like Receptors (TLRs)

Toll-like receptors (TLRs), major molecules linking the innate and adaptive immunity, are germ line-encoded pattern-recognition receptors (PRRs), detecting specific pathogen-associated molecular patterns (PAMPs) and thereby promoting immune cells' activation [69, 70]. G-MSCs may interact with their inflammatory environment via toll-like receptors (TLRs). A recent study outlined a distinctive G-MSCs' TLRs expression profile [71]. In basic medium, G-MSCs expressed TLRs 1, 2, 3, 4, 5, 6, 7, and 10. The inflammatory medium significantly upregulated TLRs 1, 2, 4, 5, 7, and 10 and diminished TLR 6 expression. Whether this differential up/downregulation of the TLRs is reflective of an increased/decreased ability to respond to the respective ligands remains to be explored. The described TLRs' expression profile of G-MSCs in inflamed and uninflamed conditions could impact their therapeutic potential in inflammatory environments in vivo [72].

### 7.2. Dendritic Cells

Dendritic cells (DCs) are major antigen-presenting cells, linking the innate and adaptive immunity [73]. Prostaglandin E_2_ (PGE_2_), a lipid mediator produced from arachidonic acid by cyclooxygenase (COX), acts on four cellular receptor subtypes (EP1–EP4), encoded by Ptger1−Ptger4 genes, causing diverse physiological actions, including pyrexia, pain sensation, and inflammation. PGE_2_ may further exert an anti-inflammatory effect, especially when binding with EP3 receptors usually present on mast cells (discussed in detail below) [74]. DCs express EP4 and its binding to PGE_2_ normally induces an IL-23 mediated proinflammatory reaction with Th-17 activation [74]. However, through PGE_2_ production, G-MSCs were reported to significantly arrest the maturation and activation of DCs, reducing their antigen presentation capacity and attenuating the inflammatory response [68]. This could be explained by an elevation/activation of the anti-inflammatory cytokine IL-10, through a PGE_2_-mediated activation of the system E prostanoid (EP) receptor/cAMP/protein-kinase-A (PKA), which phosphorylates S133-cAMP response element-binding protein (CREB), to create a docking site for the coactivator CREB-binding protein and the initiation of IL-10 transactivation. PKA activity also inhibits salt-induced kinases (SIKs), which allow the cytoplasmic retention of CREB coactivators transducer of regulated CREB activity, (TORC)/CREB-regulated transcriptional coactivator (CRTC) 2, and TORC/CRTC3, and thereby elevates IL-10 levels [75]. PGE_2_ further represses the TLR-induced cytokine induction in DCs in the absence of IL-10 [75], thereby contributing to the anti-inflammatory effect. This PGE_2_-mediated attenuation effect may be reversed through indomethacin, an inhibitor of cyclooxygenases [68].

### 7.3. Macrophages

Macrophages, essential cellular components of the innate immune response [73], can generally be categorized into M1 (proinflammatory) and M2 (anti-inflammatory) subpopulations. M2 macrophages are considered to possess anti-inflammatory properties in light of their increased production of anti-inflammatory cytokines, including IL-10, and TGF-*β* [76], which could affect T-cells (see the following). G-MSCs demonstrated an ability for the polarization of macrophages into the M2 phenotype via enhanced secretion of IL-6, IL-10, GM-CSF, and PGE_2_ [29, 33]. The immunomodulatory effect exerted by PGE_2_ is expected to be the same as described above. This in turn reduces the inflammatory response in the tissues.

### 7.4. Mast Cells

Mast cells (MCs), key cells of the innate immunity, are critical in allergic and inflammatory disorders [77]. G-MSCs demonstrate suppressive effects on specific functions of MCs in vitro and in vivo, including de novo production of the major proinflammatory cytokine TNF-*α*, from activated human mast cells (HMC-1) in a cell-cell contact-independent manner. The outlined G-MSCs-induced blockage of the de novo production of proinflammatory cytokines by MCs is alleged to be partly mediated by the tumor necrosis factor-alpha/prostaglandin E_2_ (TNF-*α*/PGE_2_) feedback axis. However, G-MSCs demonstrated no obvious inhibitory effects on MCs' degranulation in vitro. In vivo, however, G-MSCs' administration suppressed MCs' degranulation. The described inhibitory effects were dependent on the COX_2_/PGE_2_ pathway and mediated by PGE_2_-EP3 receptors [78], suggesting collectively that the TNF-*α*/COX_2_/PGE_2_ axis constitutes a negative feedback loop in the crosstalk between G-MSCs and MCs [68].

## 8. Effects of G-MSCs on the Acquired Immune System

*Effects of G-MSCs on T-Cells. *G-MSCs have been shown to exhibit a powerful dose dependent suppressive effect on the cellular proliferation and activation of human peripheral blood mononuclear cells (PBMC) stimulated either by phytohemagglutinin (PHA) [25] or by allogenic lymphocytes in a mixed lymphocyte reaction (MLR) [15, 20]. G-MSCs appear to possess the ability to suppress the proliferation of mitogen-activated lymphocytes in vitro [18, 20, 29]. The G-MSCs' suppressed PHA-dependent T-lymphocyte proliferation and activation occur via upregulation in IL-10 and downregulation tryptophan secretion in a cell-cell contact dependent and independent manner, seemingly mediated via indoleamine 2, 3-dioxygenase. (IDO) [25, 33]. The inflammatory cytokine INF-*γ*, secreted by activated T-lymphocytes in the coculture system, is assumed to act hereby as a feedback signal between G-MSCs and T-cells [25]. Additionally, findings from both in vitro and in vivo studies showed that G-MSCs could significantly inhibit Th17 cells and simultaneously promote the expansion of CD4^+^CD25^+^FoxP3^+^ regulatory T-cells (Tregs), a cell type that has been recognized to play an important role in controlling autoimmunity [79–82]. The mechanism underlying it is believed to be mediated through a TGF-*β* dependent mechanism, involving M2 macrophages, following the uptake of apoptotic T-cells. The latter effect is induced through the Fas-Ligand (FasL) secreted by the G-MSCs, a type-II transmembrane protein, belonging to the TNF family, which through binding with its receptor induces T-cell apoptosis [42].

Collectively, G-MSCs' induced immunomodulation [20, 29, 68, 83, 84], through a complex interplay with various inflammatory cells and molecules, represents a promising and an effective treatment perspective for various inflammatory and autoimmune diseases.

## 9. G-MSCs' Cell Delivery Strategies

Providing a suitable microenvironment for MSCs' delivery, proliferation, and differentiation in the presence of exogenous stimuli and growth factors is a critical step toward successful clinical applications [47, 85]. As a fundamental part of the tissue engineering triad, consisting of cells, biomolecules, and scaffolds, cell delivery vehicles or scaffolds play an important role in the in vivo performance of MSCs and could influence the outcome of any regenerative therapy [86]. A variety of cell delivery approaches currently exist for G-MSCs' application, including scaffold-free direct local or systemic injection for homing [30, 34, 41], cell sheet engineering [87], and scaffold-augmented G-MSCs' transplantation [22, 38, 39, 47, 88, 89].

For mandibular and calvarial critical size defect reconstruction, G-MSCs were seeded in a collagen gel scaffold [22]. A periodontal regeneration study, seeding G-MSCs on collagen and inorganic bovine bone matrix, demonstrated that the cells attached and spread on both scaffold types prior to their transplantation into the experimental animals [89]. Multiple studies outlined the positive regenerative effect of a RGD (arginine-glycine-aspartic acid) tripeptide, vital peptides for cellular recognition, and attachment via integrins, enclosing alginate scaffold. The scaffold provided inward flux of nutrients and sufficient levels of oxygen, mimicked the natural cell-interactive function of the extracellular matrix (ECM), and provided a favorable physiochemical microenvironment with ligands, which specifically bind with G-MSCs' receptors. Encapsulated G-MSCs differentiated into osteogenic and adipogenic tissues in vitro, demonstrating that the encapsulation process did not negatively affect their stem/progenitor cells properties [38, 39, 47].

A study incorporated G-MSCs together with interleukin-1 receptor antagonist (IL-1ra) in a hyaluronic acid based synthetic hydrogel extracellular matrix (HA-sECM) and demonstrated successful cell inclusion, via SEM examination, as well as a controlled short-term IL-1ra release prior to transplantation into an experimental periodontitis model in vivo. On transplantation G-MSCs/HA-sECM construct demonstrated a remarkable periodontal regenerative potential [88].

Recently, G-MSCs were seeded on tetracycline-loaded silk fibroin membranes (TC-SFMs). Significantly higher cell viability was noted with 1% and 5% TC-SFMs. The morphology of G-MSCs on 0% and 1% TC-SFMs showed spindle shaped cells and at 10% TC-SFMs G-MSCs appeared spheroidal. G-MSCs cultured on 1% and 5% TC-SFMs showed higher proliferation and osteogenic potential and osteogenic gene expression for Runx2, Col-I, and BSP than G-MSCs on 10% TC-SFM [90].

The further developments of suitable G-MSCs' delivery vehicles/scaffolds, of their mechanical properties, their consistency, and their controlled resorption/tissue replacement, and of the incorporation and controlled release of biological molecules in a biomimetic manner remain all aspects for vital future improvement and research in the field of G-MSCs' transplantation.

## 10. G-MSCs' In Vitro Preconditioning

Numerous innovative and traditional biological agents as well as culturing conditions, including enamel matrix derivative (EMD), traditional oriental herbal medicines, vitamin C, Risedronate, and hypoxia, have recently been tested for their preconditioning effect in vitro in an attempt to improve the cellular properties and regenerative treatment outcome of G-MSCs in vivo.

### 10.1. Enamel Matrix Derivative (EMD)

Emdogain is a commercially available enamel matrix derivative (EMD) [91], comprised of a mixture of hydrophobic enamel matrix proteins, nearly 90% of which is amelogenin, along with other enamel matrix proteins, such as amelin, ameloblastin, enamelin, and tuftelin [92], in a antimicrobial propylene-glycol-alginate (PGA) carrier. During tooth germ development, EMD is produced by the epithelial root sheath of Hertwig and plays a crucial role during root cementogenesis and during the development of the periodontal apparatus anchoring the root cementum to the surrounding alveolar bone via Sharpey's fibers [93]. In vitro studies reported on the aptitude of EMD to induce proliferation, migration, adhesion, mineralization, and differentiation as well as the increased collagen and protein production in periodontal ligament, dental follicle, and alveolar bone proper-derived stem/progenitor cells [94–97]. In vitro EMD preconditioning enhanced G-MSCs' proliferation. EMD further induced their osteogenic differentiation, with an amplified mRNA expression of Cbf*α*-l (a transcription factor of the runt-domain gene family), ALP (the early marker of osteogenic differentiation), and OC (the specific late marker of osteogenic differentiation and the major noncollagenic protein of the bone matrix) as well as an increased calcified nodule formation [40].

### 10.2. Traditional Oriental Herbal Medicines

Traditional oriental herbal medicines used in China, Japan, and Korea as* Asiasari radix* (*A. radix*),* Cimicifugae rhizoma,* and* Angelicae dahuricae* radix have been tested for their effect on G-MSCs in vitro.* A. radix*, commonly used in the treatment of dental diseases, including toothache and aphthous stomatitis, negatively influenced the viability and altered the morphology of G-MSCs in vitro [98]. Similarly,* Cimicifugae rhizoma*, commonly used as an anti-inflammatory, analgesic, and antipyretic remedy, negatively influenced the viability of the G-MSCs, especially at high concentrations, reducing cell number and CCK-8 values as well as altering their morphology from spindle to round shaped [99]. In contrast,* Angelicae dahuricae* radix, also an anti-inflammatory, analgesic, antipyretic, and antioxidant remedy, showed no effect on cell viability or morphology of G-MSCs [100]. Studies on these agents are still at an early stage, making it hard to draw a conclusion on the mechanism of action, the feasibility, and value of these herbal remedies in G-MSCs' preconditioning.

### 10.3. Vitamin C (Ascorbic Acid)

Vitamin C (ascorbic acid (AA)) is a commonly used vitamin with antioxidant properties. Earlier studies confirmed that AA, an essential agent in stem/progenitor cells' proliferation, is characterized by its ability to trigger pluripotent markers' expression in both adult and embryonic stem cells [101, 102]. G-MSCs cultured in various concentrations of AA (10–250 *μ*M) showed increased cell proliferation, significantly reducing the S and G2/M cell cycle time in a dose dependent manner. However, with AA concentrations higher than 250 *μ*M (the cell-toxicity threshold), AA could intoxicate G-MSCs and drive them to apoptosis [27]. The increased cell proliferation effect could be attributed to the fact that AA upregulates the expression of multiple proliferation-related genes, comprising Fos, E2F2, Ier2, Mybl1, Cdc45, JunB, FosB, and Cdca5 as well as the mRNA expression of HGF, IGFBP6, VEGF, bFGF, and KGF [101].

AA-treated G-MSCs at concentrations below the defined cell-toxicity threshold showed significantly higher expression of the regenerative markers SSEA-3, Sox-2, Oct-3/4, Nanog, and TRA-1-60 and maintained the G-MSCs' phenotype, their marker expression, and their cell differentiation capacity [27]. Similar reports showed that AA plays a crucial role in inducing a pluripotent state in mouse embryonic stem cells through the modulation of micro-RNA expression [103]. Further reports suggested that AA can enhance somatic reprogramming to produce pluripotent stem cells [102]. The underlying mechanism is postulated to be related to the increase of promoter activity of pluripotent genes and enhancer protein levels [28].

Interestingly, despite the demonstrated pluripotency-inductive effect in vitro, AA preconditioned G-MSCs showed no tumor formation when transplanted in athymic mice in vivo [27]. The potential of AA and other biomolecules to affect the MSCs' potency opens a new perspective in G-MSCs' research.

### 10.4. Risedronate

G-MSCs were cultured in the presence of Risedronate (1–10 *μ*M), a nitrogen-containing bisphosphonate commonly used for the prevention and treatment of postmenopausal and corticosteroid-induced osteoporosis. The drug is reported to reduce bone turnover and decrease resorption, chiefly through its effects on osteoclasts, with no undesirable effect on cortical porosity, thickness, or cancellous bone volume [104]. G-MSCs treated with Risedronate showed notable negative alterations in the morphology of the cells with fewer, rounder cells, alterations in the cytoskeletal organization, and reduced viability with decreased CCK-8 values [105].

### 10.5. Hypoxia

Hypoxia may be a promising preconditioning agent to promote the regenerative/reparative potential of G-MSCs in cell-based therapies. 2% hypoxic stimulation promoted the immunomodulatory properties of G-MSCs, through enhancing their suppressive effects on peripheral blood mononuclear cells (PBMCs), inhibiting their proliferation and increasing their apoptosis. This effect was attributed to the expression of FasL, which through its binding with its receptor induces cell apoptosis, by G-MSCs in the hypoxic environment [42].

Systemically infused G-MSCs enhanced skin wound repair in vivo and a 24-hour hypoxic preinfusion stimulation significantly supported their reparative capacity. The delivered G-MSCs inhibited the local inflammation of the injured skin through inflammatory cells' suppression, reducing TNF-*α* and increasing the anti-inflammatory cytokine IL-10. These effects were reinforced by hypoxia [42]. The results point at the positive potential of possible hypoxic preconditioning of G-MSCs, prior to their therapeutic application. Further studies are needed to validate these effects and develop, in light of the obtained results, enhanced standardized G-MSCs' culturing protocols.

## 11. Experimental Therapeutic Applications of G-MSCs

### 11.1. Skin Wound Repair

Considering the characteristically observed scarless gingival intraoral wound healing properties, G-MSCs have become an exciting alternative for tissue engineering approaches, aiming at enhanced wound repair in extraoral tissues, originally branded, in secondary healing intentions, by scar formation [11, 13]. The utility of treating wounds with G-MSCs has recently been demonstrated through their systemic infusion for wound repair in a mouse model [106]. Besides a local enrichment in multipotent and self-renewing G-MSCs at the wound site, one of the mechanisms by which G-MSCs were assumed to improve repair is via their modulation of the local inflammatory response. As discussed above, G-MSCs are proposed to promote polarization of macrophages toward the regenerative (M2) phenotype, causing a rise in the level of anti-inflammatory IL-10 and a concomitant decrease in the expression of M1-cytokines (TNF-*α* and IL-6), thereby attenuating the local inflammation, promoting angiogenesis, and significantly enhancing wound repair [106]. The previously described immunomodulatory, in addition to the tissue-regenerative effect of G-MSCs, could bring about the observed outstanding wound repair attributes.

### 11.2. Tendon Regeneration

Tendon injuries are common in sports and in everyday life. The successful repair or regeneration of the injured tendon remains a clinically challenging task, especially in light of the reduced blood supply and cellular activity in the tendon areas of the human body. Earlier studies reported on the positive effect of the application of MSCs in tendon repair and regeneration [107, 108]. G-MSCs encapsulated in an injectable and biodegradable TGF-*β*3-loaded RGD-coupled alginate hydrogel microspheres scaffold (see scaffolds description above) were tested as an alternative treatment modality for tendon regeneration. Following a subcutaneous encapsulated G-MSCs' transplantation into immunocompromised mice, ectopic de novo tendon regeneration was observed, comparable to that induced by BM-MSCs. The results were evident by a positive immunohistochemical staining of the tissues using antibodies against the specific tendon markers Tenomodulin (Tnmd), Eya1, Eya2, and Scleraxis (Scx), confirming the regenerative capacity of the encapsulated G-MSCs [38]. Further studies are needed to validate the observed tendon repair/regeneration effect.

### 11.3. Bone Defects Regeneration

Multiple studies outlined the positive potential of G-MSCs in the field of MSCs-based bone reconstruction [18, 22]. eGFP-labelled G-MSCs seeded on Col-I gel implanted into mandibular (5 × 2 × 1 mm) as well as critical size calvarial defects (5 mm in diameter) in rats showed bone reconstruction potential over 2 months [22]. Transplanted G-MSCs encapsulated in a RGD-coupled alginate microencapsulation system were tested for their regenerative ability in 5 mm diameter critical size calvarial defects in immunocompromised mice. G-MSCs, despite showing reduced osteogenic differentiation capability, were able to repair the critical size defects. These newly formed bony tissues were immune-positive for Runx2 and OC antibodies [39]. G-MSCs preconditioned in an osteogenic differentiation medium showed induction of Runx2, ALP, and osterix expression, with mineralized nodules formation. When transplanted into C57BL/6J mice with mandibular bony defects via the tail vein, G-MSCs homed to the bone defects and promoted bone regeneration [41]. All of these results combined confirm a clear bone regenerative capacity by G-MSCs.

### 11.4. Periodontal Regeneration

G-MSCs are considered a promising and readily available cell source for periodontal tissue regeneration, including the reestablishment of functional tooth cementum, periodontal ligament, and alveolar bone. In an earlier study, porcine free gingival margin derived stem/progenitor cells isolated via a minimally invasive procedure and magnetically sorted, employing anti-STRO-1 antibodies and delivered on collagen or inorganic bovine bone matrix, showed a remarkable periodontal regenerative capacity in vivo [89]. This result evidently challenged the classical periodontal compartmentalization theory, declaring that the gingiva does not contribute to periodontal regeneration and that it should be excluded via guided tissue regeneration (GTR) barriers [109], showing that its connective tissue harbored multipotent stem/progenitor cells with a significant periodontal regenerative potential.

In a further study, GFP-labelled G-MSCs' cell sheets cultured in the medium supplemented with 100 mg/mL AA were employed for periodontal regeneration in a class III furcation defects dog model. The transplanted G-MSCs significantly enhanced the regeneration of the damaged periodontal tissues, including the alveolar bone, cementum, and periodontal ligament [87].

Recently, periodontal regenerative potential of G-MSCs combined with a short-term releasing IL-1ra hyaluronic acid based hydrogel synthetic extracellular matrix demonstrated a remarkable periodontal regenerative potential in a porcine experimental periodontitis model in vivo, with newly formed bone, cementum, and periodontal ligament fibers [88].

### 11.5. Peri-Implantitis

Peri-implantitis, one of the most serious medium- and long-term complications following dental implants oral rehabilitation, is characterized by bacterial destructive inflammatory changes in the tissues surrounding and supporting the dental implant [110]. G-MSCs encapsulated in a silver lactate- (SL-) containing RGD-coupled alginate hydrogel scaffold demonstrated antimicrobial properties against* Aggregatibacter actinomycetemcomitans* (Aa) on the surface of titanium disc, mimicking a peri-implantitis model in vitro, while maintaining the G-MSCs' proliferation and osteogenic differentiation capacity. Silver ions, effectively released from the SL-loaded alginate microspheres for up to two weeks, were responsible for the antibacterial activity and the effect was dose dependent [111]. This in addition to the previously described G-MSCs' anti-inflammatory potential (see the above) could make them attractive agents in peri-implantitis treatment. Further studies are needed to explore this promising therapeutic potential in vivo.

### 11.6. Antitumor Effect

Tongue squamous cell carcinoma (TSCC) is presently the most prevalent type of oral cancer [112]. It clearly affects the life quality of the affected patients with malfunction of mastication, speech, and deglutition. Despite recent improvements in diagnostic techniques and therapeutic approaches, the number of deaths linked to TSCC increased by over 10% during the past 5 years [113]. G-MSCs therapeutic application could provide a new hope for its management. G-MSCs showed the ability to migrate towards TSCC cell lines (Tca8113 and Cal27) in an in vitro transwell cell-migration-assay, inducing tumor cell necrosis and apoptosis. Tumor necrosis factor-related apoptosis-inducing ligand (TRAIL), a member of the TNF superfamily, is a type 2 transmembrane death ligand that causes apoptosis of transformed cells, but not in most of the normal cells [114]. TRAIL-transduced G-MSCs were administered to nude mice locally and systemically (mixed injection with tumor cells and tail vein injection). The transduced cells migrated toward TSCC in a large quantity and homed efficiently, reducing or even inhibiting TSCC growth, especially when the ratio of TRAIL-transduced G-MSCs to tumor cells was 1 : 1 [30]. Taking into account the clinical difficulties commonly encountered, as the unexposed tumor sites and the difficulty of topical administration of drugs, the proposed approach could present a future promising solution for local therapeutic delivery of biomolecules and cell.

### 11.7. Oral Mucositis

One of the major side effects of head and neck anticancer radio- and chemotherapy, affecting patients' life quality, is the resultant oral mucositis, secondary to basal cell layers damage and the subsequent impaired regenerative capacity of the oral epithelium. Anticancer therapy-induced oral mucositis represents a challenging and painful clinical situation showing a persistent oral wound characterized by atrophy, erythema, ulceration, and, eventually, loss of the mucosal barrier functions [115].

Employing an in vivo murine model of chemotherapy-induced oral mucositis, spheroid-derived G-MSCs delivered systemically reserved body weight loss and promoted the regeneration of disrupted epithelial lining of the murine mucositic tongue. 3D spheroid cultures of G-MSCs expressed high levels of reactive oxygen species, hypoxia-inducible factor- (HIF-) 1 and -2a, superoxide dismutase-2 (SOD2), and manganese superoxide dismutase, which improved their resistance to oxidative stress-induced apoptosis. Spheroid cultures derived G-MSCs displayed improved cell plasticity and aptitudes to home to mucositic lesions. The relatively smaller cell sizes and increased expression of CXCR-4 by spheroid cultures derived G-MSCs facilitated their faster trafficking through the lung microvasculature and more efficient distribution into mucositis affected tissues. These effects ameliorated the chemotherapy-induced oral mucositis lesions [34] and hold a promising therapeutic potential, warranting further in-depth research.

### 11.8. Experimental Colitis

G-MSCs ameliorated dextran sulfate sodium- (DSS-) induced colitis in a mouse model. Systemic infusion of G-MSCs in experimental colitis significantly improved both clinical and histopathological severity of the colonic inflammation, refurbished the injured gastrointestinal mucosal tissues, reversed diarrhea and weight loss, and suppressed the overall disease activity. The therapeutic effect of G-MSCs was suggested to be mediated, in part, by the suppression of inflammatory infiltrates and inflammatory cytokines/mediators, the increased infiltration of regulatory T-cells, and the expression of anti-inflammatory cytokine IL-10 at the colonic sites [25]. The immunomodulatory effect of G-MSCs was further hypothesized to be associated with upregulated expression of the FasL, which plays an important role in MSCs-based immunomodulation (see the above) [36]. Additional studies are needed to further elucidate the exact mechanism underlying the described colitis-ameliorating therapeutic effect.

### 11.9. Collagen-Induced Arthritis (CIA)

G-MSCs may provide a promising therapeutic approach for the treatment of patients suffering from rheumatoid arthritis and other autoimmune diseases. G-MSCs significantly attenuated inflammatory arthritis in a collagen-induced arthritis (CIA) model. The therapeutic effects of G-MSCs depended mainly upon CD39/CD73-induced signals and partially upon the induction and expansion of Tregs (see the above). G-MSCs may suppress CIA directly in a CD39 or CD73 dependent manner. However, G-MSCs may also exert an indirect suppressing effect via promoting Tregs' production through CD39 and CD73 signaling, as was demonstrated by the fact, that G-MSCs pretreatment with CD39 or CD73 inhibitors abolished G-MSC-mediated Tregs' upregulation [116].

### 11.10. Contact Hypersensitivity

Systemic infusion of G-MSCs prior to sensitization and challenge phase dramatically suppressed hapten-induced murine contact hypersensitivity (CHS), an experimental model for human allergic contact dermatitis (ACD), one of the prevalent skin diseases worldwide. G-MSCs' infusion modulated the function of multiple innate and adaptive immune cells through the COX/PGE_2_ pathway, resulting in a decreased infiltration of DCs, CD81 T-cells, Th-17, and MCs, a suppression of a variety of inflammatory cytokines, a reciprocal increased infiltration of Tregs, and an expression of IL-10 at the regional lymph nodes and the allergic contact areas. G-MSCs further blocked de novo synthesis of proinflammatory cytokines by MCs via PGE_2_-dependent mechanisms [68] (see the above). All of these effects combined account for the hypersensitivity ameliorating effect of G-MSCs.

## 12. Conclusion and Outlook

The human gingival connective tissue provides a readily accessible as well as easily obtainable and renewable source of multipotent postnatal stem/progenitor cells for cellular approaches in different tissue repair/engineering/regeneration performances. The striking positive attributes of G-MSCs make them attractive cellular sources in the field of tissue engineering. G-MSCs show remarkable tissue reparative/regenerative potential, noteworthy immunomodulatory properties, and primary experimental therapeutic applications of G-MSCs are very promising, pointing at future biologically based therapeutic techniques, being potentially superior to conventional clinical treatment modalities.

However, numerous biological and technical challenges need to be addressed prior to considering transplantation approaches of G-MSCs a clinical reality in humans. Of prime importance remain the further optimization of techniques for cellular integration and propagation in apt biocompatible scaffolds and the improvement of their properties for clinical handling. Potential ex vivo karyotypic instability with possible gene mutations in prolonged cell-expansion-cultures remains currently hazardous outcome possibilities. Presently, the different inductive/differentiation/growth factors and cellular processes activated during stem/progenitor cells' self-renewal and differentiation are not satisfactorily illuminated. Most of our present understanding and elucidation models stem from in vitro cell culture and in vivo animal models, which do not entirely translate to human clinical situations. Finally, in view of our current knowledge gaps of tissue development processes, deeper understanding of biological processes is required, before reliable biologically based regenerative therapies become a clinical reality.

## Figures and Tables

**Table 1:** Human G-MSCs isolation protocols.

Protocol number	Tissue culture method	Study
(I)	(1) Collected tissue incubated overnight with 2 mg/mL dispase at 4°C overnight to separate epithelium (2) The minced tissues are digested in 4 mg/mL collagenase IV for 2 h at 37°C (3) Cell filtered through 70 *µ*m strainer (4) Cells seeded out (5) Single-cell cloning	[25]

(II)	(1) Tissue mincing (2) Tissue digestion in 0.1% collagenase and 0.2% dispase for 15 min at 37°C (3) Discarding of the first cell fraction containing some epithelial cells (4) Tissues are that further incubated with enzyme solution for 5, 10, and 15 min and all cell fractions that are pooled (5) Cells seeded out in tissue culture flasks	[18]

(III)	(1) Tissues digested with 0.4% dispase for 30 min at 37°C followed by collagenase type I (0.66 mg/mL) for 50 min (2) Cell filtered through 70 *µ*m strainer to single-cell suspensions (3) Single-cell cloning	[20]

(IV)	(1) The minced tissues are digested in 3 mg/mL collagenase and 4 mg/mL dispase for 2 hours at 37°C (2) Cell filtered through 70 *µ*m strainer (3) Single-cell suspension plated at a concentration of 60 cells/cm^2^ (4) Selection of single-cell-derived colonies	[23]

(V)	(1) Tissue deepithelized under magnification and cut in small pieces (2 × 2 mm) and rinsed (2) Tissue placed in dry culture flasks to adhere for 30 min then medium slowly added (3) Flasks incubated for cells to grow out (4) STRO-1 magnetic cell sorting	[19]

(VI)	(1) The minced tissues are digested in 2 mg/mL collagenase and 1 mg/mL dispase for 30 min (2) Discarding of the first cell fraction containing some epithelial cells (3) Tissues that are further incubated with same enzyme solution for 90 min at 37°C (4) Cell filtered through 70 *µ*m strainer (5) Cells seeded out	[24]

**Table 2:** Multilineage induction protocols.

Differentiation direction	Inductive medium composition
Osteogenic	*α*-MEM, 15% FCS, 100 *µ*g/mL streptomycin, 1% amphotericin, 0.1 *μ*M dexamethasone, 10 mM *β*-glycerophosphate, and 50 *μ*g/mL ascorbic acid

Adipogenic	*α*-MEM, 15% FCS, 100 *µ*g/mL streptomycin and 1% amphotericin, 1 *μ*M dexamethasone, 10 *μ*g/mL insulin, 100 *μ*g/mL 1-methyl-3-isobutylxanthin, 60 *μ*M indomethacin, and 4 mM L-glutamine

Chondrogenic	*α*-MEM, 100 *µ*g/mL streptomycin and 1% amphotericin, 10 ng/mL TgF-*β*, 0.1 *μ*M dexamethasone, 50 *μ*g/mL ascorbic acid, 10 *μ*g/mL insulin, and 1% ITS 100x

Neuronal	(I) Cells cultured on chamber slides coated with poly-D-lysine/laminin, cultured in DMEM/F12 with 10% FBS, 1 × N-2 supplement, 100 U/mL penicillin and 100 *µ*g/mL streptomycin, 10 ng/mL fibroblast growth factor 2, and 10 ng/mL epidermal growth factor (II) Cells cultured on chamber slides coated with poly-D-lysine/laminin, cultured in DMEM/F12 with 125 ng/mL basic fibroblast growth factor (bFGF), 1000 unit/mL leukemia inhibitory factor, and 4 mM forskolin

Endothelial	Cells cultured in 8-well chamber slides precoated with fibronectin and cultivated in the presence or absence of endothelial growth medium 2

**Table 3:** Major surface markers expressed on G-MSCs.

Study	CD13 %	CD14 %	CD29 %	CD31 %	CD34 %	CD38 %	CD44 %	CD45 %	CD54 %	CD73 %	CD90 %	CD105 %	CD117 %	CD146 %	CD166 %	SSEA-4 %	STRO-1 %	HLA-DR %	Oct-4	Nanog
Zhang et al., 2009 [25]			99.8					0.1		99.9	100	29.9		7.1		36.9	18.3		+	
Tomar et al., 2010 [18]			78.74		3.37		95.25	3.21		98.03	98.32	97.16								
Fournier et al., 2010 [17]			100		0		100	0		100	100	100		3–17			35	0		
Mitrano et al., 2010 [15]	99.48				0.95	0.25	99.4	0.85	0.80	98.98	99.52	96.1								
Tang et al., 2011 [20]			82.4		0.3		90	0.5			76.4	92.5		93.3			75.6			
Wang et al., 2011 [22]			99.98		0.01			0.41			92.87	34.75					17.89			
Zhang et al., 2012 [33]			94.7		1.23		79.0			80.4	98.3	41.1		10.8		14.2	13		+	+
Zhang et al., 2012 *Spheriod* [34]			67.5		0.6		44.8			65.4	80.0	10.8		9.5		14.7	25.2		+	+
Yang et al., 2013 [35]			100	1.3				1.9			99.9	97		55.2			16.3			
Xu et al., 2013 [36]					0.03		98.03	0.14		47.47	90.06	73.59	0.08							
El-Bialy et al., 2014 [26]		~2			~1			~1		~65	~50	~45								
El-Sayed et al., 2015 [19]		0.1			0.07			0.12		95.11	99.33	97.71		8.47			31.64			
Gao et al., 2014 [28]			13.4		1.8			1.6			99.4	99.5		8.5			10.0			
Gay et al., 2014 [37]												+				+			+	+
Moshaverinia et al., 2014 [38]					−							+		+						
Moshaverinia et al., 2014 [39]					−									+	+					
Wu et al., 2014 [40]				2.28	1.67			0.53		99.76	99.44	99.02					8.05	0.19		
Xu et al., 2014 [41]			98.17				99.43			96.71		90.85					20.31			
Jiang et al., 2015 [42]			94.5					3.9			92	25.5								
Jin et al., 2015 [24]		0.05			0.07		99.94	0.02		99.47	99.84	94.96		+	+	+	+			
van Pham et al., 2016 [27]		−			−		+	−		+	+	+						−		
Yin et al., 2016 [43]																+			+	
